# A Global Comparison of Liver Transplant Drug Pricing: US Versus Other G7 Countries and Australia

**DOI:** 10.1097/TXD.0000000000001805

**Published:** 2025-05-12

**Authors:** Leah Yao, Xiaohan Ying, Arun B. Jesudian, Robert S. Brown, Stephen E. Congly

**Affiliations:** 1 Department of Medicine, New York-Presbyterian/Weill Cornell Medical Center, New York, NY.; 2 Department of Medicine and Gastroenterology, New York-Presbyterian/Weill Cornell Medical Center, New York, NY.; 3 Department of Medicine and Gastroenterology, University of Calgary Cumming School of Medicine, Calgary, AB, Canada.

## Abstract

**Background.:**

A major contributor to increased healthcare spending in the United States is drug pricing, as US drug prices are nearly 3× higher than those in other countries. This cross-sectional study aimed to investigate current global price differences in liver transplant medications and provide potential cost savings for Medicare Part D if international reference pricing was adopted.

**Methods.:**

Publicly available drug formularies for Canada, the United Kingdom, Japan, France, Germany, Italy, and Australia were used to collect 2024 prices for 8 commonly used liver transplant medications (mycophenolate mofetil, mycophenolate sodium, sirolimus, tacrolimus, Advagraf, Envarsus, everolimus, and cyclosporine). US prices were obtained from UptoDate’s 2024 listed representative average wholesale prices and Medicare Part D’s drug prices from 2022.

**Results.:**

The average US wholesale price and Medicare spend for originator liver transplant medications were 4.1× and 6.8×, respectively, the average originator prices in G7 countries and Australia. Adopting the average global originator drug price per dose may lead to an estimated $26 141 463 in cost savings per year to Medicare. The average US wholesale price and Medicare spend for generic liver transplant medications were 2.76× and 3.75×, respectively, the global average generic prices. Adopting the average global generic drug price per dose may lead to an estimated $363 538 474 in cost savings per year to Medicare.

**Conclusions.:**

This study demonstrates the significantly greater financial burden that liver transplant patients in the United States face compared with liver transplant patients in 7 other major industrial countries. Future adoption of international reference pricing may help bridge this pricing disparity.

Healthcare spending in the United States has continued to rise drastically, with a major contributor being drug pricing.^1^ US prescription drug prices have been found to be nearly 3× higher than those in other countries.^2^ Outside the United States, other G7 countries and Australia use a centralized federal body that directly negotiates with pharmaceutical manufacturers to set drug prices.^3^ Price regulation strategies such as external reference pricing and cost-effectiveness analysis are widely implemented.^3,4^ In the United Kingdom, France, Australia, and Japan, the drugs’ therapeutic value is also often considered in setting reimbursement rates.^3^ In the United States, there is no universal price control to regulate drug prices. Drug prices are market driven, directly set by pharmaceutical companies with additional discounts or rebates negotiated by payers such as pharmacy benefit managers or insurance companies.^4^ Only recently under the Inflation Reduction Act of 2022 has Medicare been allowed to directly negotiate prices for certain medications, which may lead to substantial savings in the future.^5,6^ Studies have predicted that the adoption of an international reference pricing tool could also lower US drug spending by 52%, or $83.5 billion annually.^7^

In the United States, prescription drug coverage is paid for by a combination of public healthcare programs (Medicaid or Medicare), private insurance, and out-of-pocket payments by patients. Medicare is a US federal health insurance program for those over the age of 65 (as well as those with end stage renal disease and disabilities) that includes prescription drug coverage benefits. The cost of immunosuppressive therapies can pose a significant financial burden to liver transplantation (LT) recipients.^8-10^ Patients may be billed >$30 000 a year for transplant medications alone.^9^ High out-of-pocket medication costs for patients may lead to nonadherence and negatively impact post-LT outcomes such as hospital readmissions.^11^ The introduction of generic versions of immunosuppressant medications has resulted in significant cost savings for Medicare and patients; however, US transplant patients may still be facing pricing disparities compared with their global counterparts.^8^ In the other G7 countries and Australia, the majority of drug costs are covered by a public payer program with comparatively minimal or limited out-of-pocket costs for patients.^12,13^

There has been limited literature directly comparing the cost of medications related to liver disease and/or transplantation in the United States and other industrialized countries.^14,15^ This cross-sectional study aims to be the first to investigate price differences in immunosuppressive medications used for LT in the United States as compared with the other G7 countries and Australia. This study also explores potential cost savings to Medicare Part D if international reference pricing is adopted.

## MATERIALS AND METHODS

Eight LT immunosuppressive medications (mycophenolate mofetil, mycophenolate sodium, sirolimus, tacrolimus, Advagraf, Envarsus, everolimus, and cyclosporine) were chosen as they comprise the majority of drugs used for LT immunosuppression based on the literature and the United Network for Organ Sharing data. Publicly available drug formularies published by government websites for each comparison country (Canada, the United Kingdom, Japan, France, Germany, Italy, and Australia) were used to collect 2024 prices for the selected medications at varying doses. Canada is the only country in this study with regulation of drug prices on both a federal and provincial level; there is no national formulary but each province has their own formulary. Ontario was chosen as a representative example for Canada for drug pricing; overall prices for drugs are similar across the country with slight differences based on dispensing fees.^16^ Both originator (the first brand name drug on the market) and generic prices for medications were collected when available. Foreign currencies were converted to USD based on the conversion rate on January 1, 2024.

Current US drug prices were obtained from UpToDate’s listed representative average wholesale price (AWP) from manufacturers.^17^ AWP represents the fixed list price set by manufacturers prior to any negotiations with payers, discounts, or rebates. If a range of prices was provided from multiple manufacturers, the lowest price was selected for a more conservative price comparison. Medicare Part D drug prices from 2022, listed as the average spend per dosage unit weighted, were also collected from the Center for Medicare & Medicaid Services.^18^ Medicare prices reflect the amount that Medicare actually reimburses for medications, based on market-driven rates and after negotiations by prescription drug plans with manufacturers. Dosage unit refers to the drug unit in the lowest dispensable amount. Thus, for each immunosuppressive medication the price difference between the Medicare drug price and the average global drug price at the lowest dose was multiplied by the total dosage units dispensed to estimate the annual cost savings for Medicare Part D if the average global price was to be adopted. If there were fewer than 3 countries of pricing data available for a given medication, the medication was excluded from the total cost savings calculation.

This study was exempt from IRB approval.

## Results

### Average Wholesale Price

US originator immunosuppressive drug prices based on the AWP were on average 4.11× (range 1.22–10.1×) the average drug prices of G7 countries and Australia. At its lowest dose of 250 mg, mycophenolate mofetil costs $10.80 in the United States with the price range of $0.56–$2.68 among comparison countries. Similarly, mycophenolate sodium (180 mg) costs $8.14 in the US compared with $0.86–$1.73 globally, sirolimus (0.5 mg) $20.63 versus $1.96–$6.13, tacrolimus (0.5 mg) $4.31 versus $0.86–$3.91, Advagraf (0.5 mg) $3.37 versus $0.87–$3.91, Envarsus (0.75 mg) $6.03 versus $1.45–$5.71, everolimus (0.25 mg) $12.65 versus $1.25–$4.24, and cyclosporine (25 mg) $3.24 versus $0.59–$2.62 (Table 1).

**TABLE 1.** Liver transplant drug pricing by country^*a*^

Drug	Unit (mg)	The United States	Canada	France	The United Kingdom	Japan	Italy	Germany	Australia
Mycophenolate Mofetil	250	10.8^*b*^ (0.32)^*c*^	1.57 (0.28)	0.56	1.05	0.95 (0.65)	1.96 (0.98)	1.03 (0.92)	2.68
	500	21.59 (1.14)	3.14 (0.56)	1.11	0.15	N/A	3.93 (1.63)	1.83 (1.72)	2.68
Mycophenolate Sodium DR	180	8.14 (4.39)	1.51 (0.75)	0.86	1.03	N/A	1.73 (0.92)	1.02 (1.02)	0.97
	360	16.29 (9.13)	3.02 (1.51)	1.72	2.05	N/A	3.47 (1.83)	1.77 (1.02)	1.95
Sirolimus	0.5	20.63 (8.47)	N/A	1.96	2.93	N/A	N/A	6.13 (5.85)	4.00
	1	41.25 (16.95)	6.89	3.91	3.67	9.29	N/A	12.10 (11.34)	8.00
	2	82.5 (31.5)	N/A	7.80	7.34	N/A	N/A	23.84 (21.53)	16.01
Tacrolimus	0.5	4.31 (1.26)	1.53 (0.77)	0.86 (0.66)	1.58	1.74 (1.05)	N/A	3.91 (3.41)	0.86
	1	8.62 (1.64)	1.96 (0.98)	1.72 (1.30)	2.04	3.22 (1.91)	3.40326 (1.91)	5.52 (4.81)	1.72
Advagraf ER	0.5	3.37	1.68	0.87 (0.83)	0.91	N/A	N/A	3.91 (3.41)	1.70
	1	6.73	2.14	1.73 (1.64)	1.82	N/A	3.42	5.81 (4.80)	1.72
	3	N/A	6.43	5.15 (4.89)	5.47	N/A	10.23	16.31 (15.60)	8.97
	5	33.65	10.74	8.56 (8.13)	6.80	N/A	17.11	22.48 (19.54)	17.04
Envarsus	0.75	6.03	1.59	1.45	1.88	N/A	N/A	5.71	N/A
	1	8.04	1.98	1.83	2.51	N/A	3.11	6.34	N/A
	4	32.15	7.93	7.67	10.03	N/A	12.44	20.75	N/A
Everolimus	0.25	12.65 (10.01)	N/A	1.45	N/A	N/A	3.04	4.24 (3.71)	3.45
	0.5	25.34 (20.02)	N/A	1.83	N/A	N/A	N/A	8.75 (7.56)	6.02
	0.75	37.98 (30.03)	N/A	7.67	N/A	N/A	9.14	11.47 (10.67)	18.07
	1	50.58 (40.06)	N/A	N/A	N/A	N/A	N/A	18.71 (14.5)	25.93
Cyclosporine	25	3.24 (2.1)	0.59	0.75	0.78	0.81 (0.46)	1.24 (0.55)	1.17 (1.17)	2.62
	50	2.74	1.16	1.50	1.53	1.37 (0.86)	2.41 (1.08)	2.23 (2.03)	5.45
	100	12.86 (8.4)	N/A	2.98	2.90	N/A	4.49 (2.02)	4.28 (4.37)	11.11

^*a*^Formularies used: Australia: Pharmaceutical Benefits Scheme; Canada: Ontario Drug Benefit Formulary; France: Public Drug Database; Germany: Drug Directory; Italy: National Pharmaceutical Formulary; Japan: National Health Insurance Drug Price List; UK: British National Formulary.

^*b*^All prices are listed in USD

^*c*^Generic drug prices are listed in parentheses.

N/A, not applicable, no price available.

US generic immunosuppressive drug prices based on the AWP were on average 2.76× (range 0.47–6.01×) the average generic prices in comparison countries. At its lowest dose of 250 mg, mycophenolate mofetil costs $0.32 in the United States with the price range of $0.28–$0.98 globally. Similarly, mycophenolate sodium (180 mg) costs $4.39 versus $0.75–$1.02, tacrolimus (0.5 mg) $1.26 versus $0.66–$3.41, and cyclosporine (25 mg) $2.10 versus $0.46–$1.17 globally.

### Medicare Pricing

Medicare’s average spend per dosage unit for originator immunosuppressive medications was on average 6.8× (range 3.9–10.77×) the average lowest dose of originator drug prices of G7 countries and Australia (Table 2). The estimated annual cost savings for originator immunosuppressive drugs is calculated to be $26 141 463 if the average global originator price per dosage unit is adopted as a reference price for Medicare.

**TABLE 2.** Medicare spend versus average global pricing for liver transplant drugs

Drug	Type	Avg Medicare Spend, USD^*a*^	Avg Global Price, USD^*b*^
Mycophenolate Mofetil	Originator	15.08^*c*^	1.40
	Generic	0.78	0.68
Mycophenolate Sodium DR	Originator	8.76	1.19
	Generic	2.70	0.89
Sirolimus	Originator	29.42	3.76
	Generic	8.41	N/A
Tacrolimus	Originator	5.91	1.78
	Generic	2.24	1.46
Advagraf ER	Originator	9.44	1.81
Envarsus	Originator	10.39	2.66
Everolimus	Originator	26.74	3.00
	Generic	14.36	N/A
Cyclosporine	Originator	3.45	0.49
	Generic	5.11	0.55

^*a*^Avg Medicare Spend: the average spend by Medicare per dosage unit weighted.

^*b*^Avg Global Price: the average price of the lowest dose of each medication across G7 countries and Australia.

^*c*^Prices listed are for the lowest dosage unit available for each medication.

N/A, not applicable.

The average spend per dosage unit for generic immunosuppressive medications for Medicare was on average 3.75× (range 1.15–9.29×) the average lowest dose generic drug prices of 5 other countries. The estimated annual cost savings for generic immunosuppressive drugs is calculated to be $363 538 474 if the average global generic price per dosage unit is adopted as a reference price for Medicare.

## DISCUSSION

**FIGURE 1.**
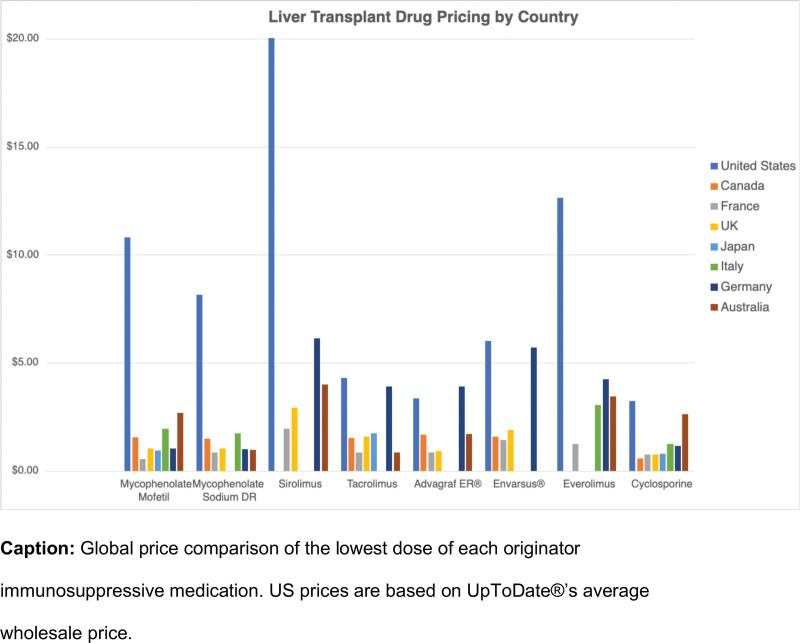
Liver transplant drug pricing by country.

This study demonstrates the significantly greater financial burden that LT patients in the United States may face compared with those residing in seven other major industrial countries. Apart from the 0.5 mg formulation of Advagraf, US AWP and Medicare prices for originator immunosuppressive medications were higher than the prices in all the comparator countries studied. Germany, Italy, and Canada tended to have higher prices than France, the United Kingdom, Japan, and Australia (Figure 1).

Only 4 of the 8 immunosuppressants had generic equivalents with prices found in 3 or more countries in the study (mycophenolate mofetil, mycophenolate sodium, tacrolimus, and cyclosporine). There was no clear pricing difference between originator drugs that do and do not have a generic equivalent. Based on the AWP data, US generic prices for mycophenolate mofetil and tacrolimus were lower than the generic prices in the other countries studied. However, based on Medicare Part D data, all four of the generic immunosuppressive medications studied were more expensive than the global average. This may confirm prior studies’ findings that the introduction of generic immunosuppressive medications has lowered drug costs for US patients, but there may still be pricing disparities when comparing the price of generic equivalents in other countries.^8^ More global data on generic drug pricing is needed to fully characterize this evolving landscape.

Drug pricing in the United States is a complex issue involving multiple stakeholders, such as federal and state governments, insurers, pharmacy benefit managers, and providers. Given several different levels of negotiation, pricing transparency is a major challenge for patients. It is a similar challenge for providers and investigators and is a limitation of this study. The AWP prices from this study do not reflect the final amount patients may pay due to manufacturer rebates, insurer negotiations, and cost-sharing.^4,19^ This may lead to higher, but more conservative estimates of the financial burden LT patients in the United States may face. The drug formulary prices from the other countries in this study also represent the manufacturer prices and not necessarily the out-of-pocket cost patients face, which may balance the direct comparison. The Medicare prices used in this study are more transparent and reflective of real-world costs than the AWP are for both branded and generic drugs. There remains difficulty translating Medicare pricing to comparison country pricing due to the lack of centralized regulatory frameworks in the United States and differences in insurance coverage models. However, with the upcoming implementation of the Inflation Reduction Act in 2026, Medicare will gain negotiating power to set drug prices and potentially achieve closer pricing parity with global peers.

Another limitation to this study is the Medicare dataset. The most recently available Medicare Part D drug pricing data is from 2022 in comparison with 2024 data from the other 7 countries used in this study. Furthermore, Medicare drug prices were only available at the lowest dosage unit, limiting direct comparison to other countries’ drug prices at varying doses. In our cost savings calculation, we directly compared Medicare drug prices to the lowest dose available of each drug in G7 and Australia countries to best reflect pricing differences.

As the annual number of LTs in the United States increase and life expectancy post-LT increases, the cost of these immunosuppressive medications will substantially impact domestic healthcare spending. This study demonstrates that utilizing international reference pricing may help bridge this pricing disparity and potentially lead to significant cost savings for Medicare and patients. Lowering immunosuppressive prices for transplant patients may improve adherence to medications and improve posttransplant outcomes, facilitating additional long-term cost reduction with fewer hospitalizations and retransplants. These potential cost savings may be redirected to bolster financial and geographic accessibility to organ transplantation programs, ultimately improving equity in transplant health.
